# Multi-omics signatures of circulating factors associated with cardiorespiratory fitness adaptations in individuals with prediabetes

**DOI:** 10.1186/s12933-026-03286-x

**Published:** 2026-07-12

**Authors:** Candela Diaz-Canestro, Kit Cheung, Enrique Roche, Jose Manuel Sarabia, Michael Andrew Tse, Aimin Xu

**Affiliations:** 1 Center for Molecular Cardiology, University of Zurich, Schlieren, Switzerland; 2 State Key Laboratory of Pharmaceutical Biotechnology, The University of Hong Kong, Hong Kong, China; 3 Department of Medicine, The University of Hong Kong, Hong Kong, China; 4 Department of Applied Biology-Nutrition, Institute of Bioengineering, Miguel Hernandez University (UMH), 03202 Elche, Spain; 5 Alicante Institute for Health and Biomedical Research (ISABIAL), 03010 Alicante, Spain; 6 CIBER for the Physiopathology of Obesity and Nutrition (CIBEROBN), Institute of Health Carlos III (ISCIII), 28029 Madrid, Spain; 7 Department of Sports Science, Sports Research Center, Miguel Hernandez University, Elche, Spain; 8 Centre for Sports and Exercise, Active Health Clinic, The University of Hong Kong, Hong Kong, China; 9 Guangdong-Hong Kong Joint Laboratory for Metabolic Medicine, The University of Hong Kong, Hong Kong, China

**Keywords:** Prediabetes, High-intensity interval training, Cardiorespiratory fitness, Proteomics, Gut microbiome

## Abstract

**Background:**

Patients with insulin resistance exhibit reduced cardiorespiratory fitness (CRF), assessed by peak oxygen consumption (VO₂peak), compared with healthy age-matched individuals. Although high-intensity interval training (HIIT) can substantially improve VO₂peak, there is considerable interindividual variability in this response. Therefore, further research is needed to elucidate the molecular mechanisms underlying the heterogeneous response of VO₂peak to HIIT in individuals with prediabetes.

**Methods:**

Proteomic analyses of serum samples, along with fecal metagenomic and targeted metabolomic profiling, were conducted in medication-naïve, overweight and obese Chinese men with prediabetes (*n* = 35; aged 24–62 years). All participants underwent a 12-week HIIT intervention, and biological samples were collected both before and after the intervention to evaluate exercise-induced alterations in circulating proteins, gut microbial composition, and metabolite profiles.

**Results:**

After 12 weeks of HIIT, mean VO₂peak increased by 0.47 L/min with individual responses ranging from 0 to 1.7 L/min. Baseline levels of short-chain fatty acid (SCFA)-producing genera, including *Prevotella* (β = 105.65, *P* = < 0.001, FDR = 0.034), *Coprococcus* (β = 50.22, *P* = 0.01, FDR = 0.39), and *Hungatella* (β = 40.72, *P* = 0.025, FDR = 0.50), were positively associated with ΔVO₂ peak. In contrast, baseline levels of the erythropoiesis-stimulating hormone erythropoietin (EPO) (β = −279.03, *P* = 0.024, FDR = 0.99) were negatively associated with ΔVO₂ peak. Exercise-induced changes in growth hormone 1 (β = 63.97, *P* = 0.04, FDR = 0.99) were positively associated with ΔVO₂ peak, whereas exercise-induced changes in BTB and CNC Homology 1 (β = −250.82, *P* = 0.01, FDR = 0.99), a repressor of heme oxygenase-1, were negatively associated with ΔVO₂ peak. In multiple linear regression analysis including clinical variables, percentage lean mass (β = 64.17, *P* = 0.0005) was the strongest variable associated with **Δ**VO₂peak. The clinical model explained 27% of the variance which increased to 37% (*P* = 0.002) upon inclusion of exercise-associated circulating factors such as EPO.

**Conclusions:**

Our findings reveal that baseline proteomic and metagenomic signatures are associated with VO₂peak adaptations. These multi-omics signatures may support the clinical implementation of personalized exercise interventions to improve CRF in individuals with prediabetes.

**Supplementary Information:**

The online version contains supplementary material available at https://doi.org/10.1186/s12933-026-03286-x.

**Research Insights**

**What is currently known about this topic?**

- Patients with insulin resistance exhibit reduced cardiorespiratory fitness compared with healthy age-matched individuals.
- There is considerable interindividual variability in the cardiorespiratory fitness response to exercise training.
- Previous studies have focused on proteomic profiles associated with VO₂peak and VO₂peak trainability in healthy populations.

**What is the key research question?**

- What molecular signatures underlie VO₂peak and its trainability in individuals with impaired glucose homeostasis?

**What is new?**

- Baseline levels of short-chain fatty acid–producing genera were positively associated with ΔVO₂peak, whereas erythropoietin was negatively associated with ΔVO₂peak.
- Exercise-induced changes in growth hormone 1 were positively associated with ΔVO₂peak, whereas exercise-induced changes in BTB and CNC Homology 1, a repressor of heme oxygenase-1, showed a negative association with ΔVO₂peak.
- Adding proteomic markers, such as erythropoietin, to the multivariable regression model increased the explained variance for ΔVO₂peak to 37%.

**How might this study influence clinical practice?**

- These findings may facilitate the clinical implementation of personalized exercise interventions to improve CRF in individuals with prediabetes.

## Introduction

Cardiorespiratory fitness (CRF), commonly assessed by peak oxygen consumption (VO₂peak), is inversely associated with cardiovascular (CV) and all-cause mortality, independent of established risk factors [1]. Extremely high CRF has been linked to the greatest survival, suggesting that an increase in VO₂peak will improve health without an apparent upper limit [2]. Individuals with insulin resistance exhibit reduced VO₂peak compared to healthy age-matched counterparts [3], and they are at higher risk of developing cardiovascular diseases compared with individuals with normal insulin sensitivity [4]. Therefore, interventions aimed at improving CRF in individuals with insulin resistance are of particular clinical importance.

Endurance exercise training (ET) is recognized as the most effective lifestyle intervention for enhancing VO₂peak, with high-volume high-intensity interval training (HIIT) often producing greater improvements than traditional ET [5]. In this regard, training dose is emerging as the primary determinant of VO₂peak improvement, and increasing the ET dose has been shown to reduce non-response in VO₂peak among healthy individuals [6]. Despite this, substantial interindividual variability in VO₂peak trainability persists, representing a critical challenge for cardiovascular prevention in metabolic diseases. This variability raises important questions regarding differential responses to HIIT, as a blunted increase in cardiorespiratory fitness may reflect a residual cardiometabolic risk despite exercise adherence. Individuals with insulin resistance often exhibit, reduced blood volume, mitochondrial impairments, chronic low-grade inflammation, endothelial dysfunction, and altered muscle perfusion [7, 8]. These factors collectively may attenuate physiological adaptations to exercise [7, 9]. On the other hand, individuals with type 2 diabetes mellitus are typically prescribed multiple pharmacological treatments, which can increase variability and make it difficult to distinguish whether observed outcomes, in particular cardiometabolic outcomes, are attributable to the medications or the intervention. In addition, certain medications, such as antihypertensive drugs, may impair hematological adaptations, thereby limiting increases in VO₂peak induced by ET [10] Consequently, it remains important to investigate VO₂peak adaptations to HIIT in medication naïve individuals with insulin resistance.

This adaptive process is largely mediated by ‘exerkines’, exercise-induced signaling molecules released by tissues such as skeletal muscle, adipose tissue, liver, bone, and the cardiovascular system that mediate inter-organ communication and contribute to the molecular and physiological adaptations to exercise [11]. In our previous study, we identified and characterized circulating proteins responsive to HIIT in individuals with prediabetes [12]. In addition, genomic and proteomic studies—such as those conducted within the HERITAGE Family Study—have primarily focused on proteomic profiles associated with VO₂peak and VO₂peak adaptations in healthy populations [13, 14]. The Molecular Transducers of Physical Activity Consortium (MoTrPAC) aims to enroll 2400 healthy participants across North America to elucidate molecular responses to ET [15]. However, a multi-omic approach is required to investigate the complex, multi-layered regulation of HIIT and decipher how the systemic release of circulating proteins coordinately modulates VO₂peak adaptations and to identify baseline molecular signatures that are associated with VO₂peak changes in individuals with prediabetes.

To address these gaps, we examined the relationships between circulating proteins, fecal metagenomics, and metabolomics with VO₂peak at baseline and following 12-week HIIT. Furthermore, multivariable linear regression models were applied to examine the associations of baseline clinical and proteomic variables with VO₂peak adaptations.

## Methods

### Experimental model and subject details

#### Ethics statement

This study was approved by the Institutional Review Board of the University of Hong Kong/Hospital Authority Hong Kong West Cluster (UW 15–370) and endorsed the principles of the Declaration of Helsinki. Written informed consents were obtained from all participants.

#### Study participants

Overweight and obese Chinese men were recruited from the local community via flyers and online advertisements, as previously described [16]. A total of 35 participants who completed the exercise intervention and had samples available at different time points were included in the proteomic analysis, whereas 20 participants were included in the metagenomic and metabolomic analyses (Supplementary Fig. 1). All participants recruited were instructed to continue their normal routine and not make any changes to their habitual physical activity and diet.

**Fig. 1:**
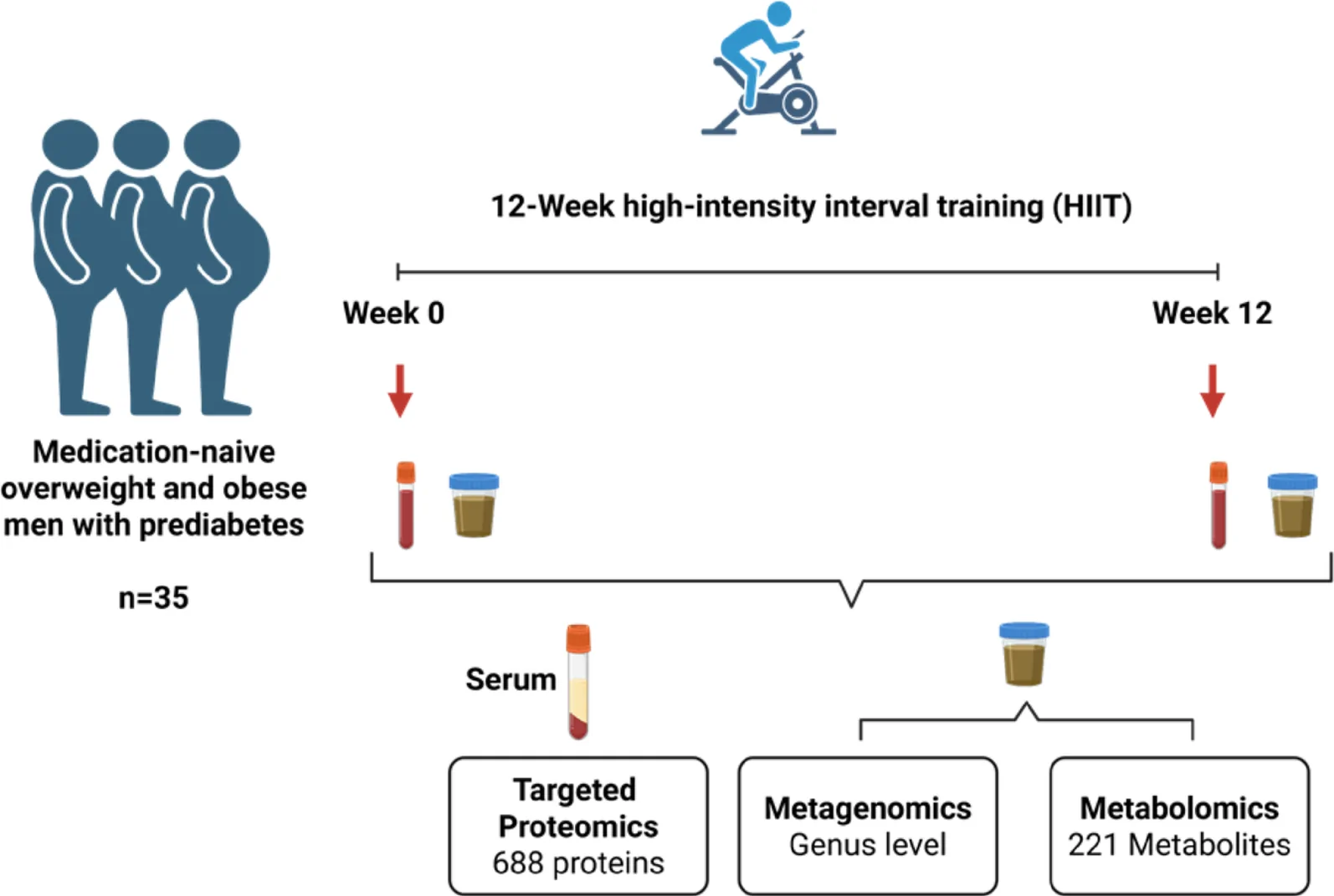
Multi-omic changes in response to high intensity interval training. Schematic diagram of the study design

### Method details

#### Participants selection

The inclusion and exclusion criteria were previously discussed [16]. Briefly, inclusion criteria comprised: non-smoking Chinese men aged 20–60 years with overweight/obesity (BMI > 23 kg/m²) and stable body weight, diagnosed with prediabetes (impaired glucose tolerance and/or impaired fasting glucose according to the American Diabetes Association guidelines criteria). Eligible individuals were not on any chronic medication. Participants were excluded if they had acute or chronic illness, inflammatory or infectious conditions, neurological, musculoskeletal, or cardiopulmonary disorders affecting exercise safety or tolerance, recent structured exercise or dietary programs, or mental illness limiting their ability to participate or understand the study. Additionally, participants who did not complete ≥ 85% of the prescribed training frequency were excluded from further analyses.

#### HIIT protocol

The 12-week HIIT consisted of three sessions per week on non-consecutive days at the Active Health Clinic, Center for Sports and Exercise, The University of Hong Kong, supervised by certified exercise specialists in a one-to-one manner, as previously described [16]. Participants were required to take part in at least 85% of all the exercise sessions for inclusion into the analysis. Each session consisted of a 70-min high-intensity combined aerobic and resistance interval training session including a 10-min warm-up, followed by participants rotating through three 10-minute stations: high-intensity treadmill intervals, high-intensity resistance and calisthenics intervals, and high-intensity stationary bike intervals. There were 3–4 min of recovery between stations. Each session ended with 10–15 min of cool-down and stretching. Intensity was adjusted using real-time heart rate telemetry, and participants were encouraged to work at 80–95% of their maximum heart rate (HRmax).

The treadmill interval station consisted of 3–4 bouts of 2 min of running at 85–95% of VO₂peak, interspersed with 30–45 s of active recovery at 50% VO₂peak. Speed and incline were adjusted individually to ensure progressive overload. The stationary bike station included 4–5 bouts of 45–60 s of high-intensity cycling at 90–95% of peak work capacity, interspersed with 60–75 s of active recovery at 30% of peak work capacity. Resistance and cadence were adjusted according to individual progression. The resistance and calisthenics station consisted of 2–3 sets of high-intensity exercises such as squats, kettlebell swings, planks, and burpees, with 30 s of rest between sets.

#### Collection of clinical data and VO₂peak

Fasting serum samples were collected at baseline and 48–72 h after the final exercise session to prevent the influence of the acute effects of exercise. Body composition was assessed via whole-body dual-energy X-ray absorptiometry (DXA) scans (Explorer S/N 91,075, Hologic Inc., Waltham, MA, USA). A graded exercise test using the Balke treadmill protocol [17] was performed on a motor-driven, electronically controlled treadmill (TrackMaster, Full Vision Inc., Newton, KS, USA) to assess VO₂peak before and after the exercise intervention. VO₂peak was assessed using a Medgraphics Ultima™ CardiO2^®^ gas exchange analysis system (MGC Diagnostics, USA). Gas exchange data were collected breath-by-breath and averaged over 30-second intervals. The system was calibrated prior to each test using certified calibration gases and a 3-L syringe according to manufacturer guidelines. Measurement error analysis showed a typical error of measurement of 1.14 mL kg⁻¹ min⁻¹ and a coefficient of variation of 2.5%, with Bland–Altman limits of agreement ranging from − 2.75 to 3.56 mL kg⁻¹ min⁻¹. After sufficient familiarization time, participants started walking at a constant speed of 5.3 km/h. The treadmill gradient began at 0% (no gradient) for the first minute, then it was increased to 2% in the second minute, after which the treadmill gradient was increased by 1% every minute thereafter until test completion. Heart rate, rate of perceived exertion (RPE) and gas analysis were measured throughout in order to assess exertion and heart rate function via measurement of oxygen consumption.

#### Olink proteomics

Antibody-based technology (Olink Proteomics AB, Uppsala, Sweden) was used for proteomics profiling in serum samples as previously described [12]. The selection of these panels was strategically aimed at identifying a broad spectrum of circulating proteins—including markers of vascular remodeling, metabolism, and systemic inflammation—to elucidate the molecular crosstalk between exercise and cardiovascular health in individuals with prediabetes. Specifically, protein biomarkers were detected via the Olink Explore 384 cardiometabolic and inflammation panels. After excluding 46 proteins that were not detected in > 50% of the samples and 3 proteins that were duplicated, the final analysis included 688 proteins (352 from the cardiometabolic panel, 333 from the inflammatory panel and 3 included in both panels, Supplementary Fig. 1). The median intra-assay coefficient of variability (CV) was 12.5%, as assessed by multiple replicates of a pooled sample included in the experiment. Values were presented as normalized protein expression (NPX) units on a log2 scale.

#### Fecal DNA sequencing and targeted metabolomics profiling

Fecal genomic DNA was sequenced on the Illumina HiSeq 4000 platform (Illumina, San Diego, California, USA; paired-end; insert size: 350 bp; read length: 150 bp) by BGI (Hong Kong S.A.R., China). Genus-level microbiome data were analyzed using a compositional data framework. Low-prevalence taxa (< 20% prevalence across samples) were excluded prior to analysis. To address zero values, a small pseudocount (1 × 10⁻⁶) was added to all taxa, followed by renormalization. We also applied a centered log-ratio (CLR) transformation, which is widely recommended for compositional microbiome data. The CLR transformation expresses each taxon relative to the geometric mean of all taxa within a sample:

$$CLR\left({x}_{i}\right)=\mathrm{l}\mathrm{o}\mathrm{g}\left(\frac{{x}_{i}}{g\left(x\right)}\right)$$

Targeted metabolomics profiling of fecal samples was performed by Metabo-Profile (Shanghai, China) as previously described [16]. Briefly, 10 mg of lyophilized feces was homogenized with 1 M NaOH and methanol, and the resulting supernatants were combined. Samples were derivatized with methyl chloroformate (MCF; Sigma-Aldrich, Stockholm, Sweden) using a MultiPurpose Sample PrepStation 2 (Gerstel, Germany), followed by the addition of internal standards. Derivatized samples were analyzed in randomized order using gas chromatography coupled to time-of-flight mass spectrometry (GC/TOFMS; Pegasus HT, Leco Corp., USA) equipped with an Rxi-5MS capillary column (30 m × 0.25 mm i.d., 0.25 μm film thickness; Agilent), as previously described by Zhao et al. [18]. Quality control (QC) samples were prepared identically to study samples and injected after every 14 samples to monitor analytical reproducibility and instrument stability. Raw data were processed using ChromaTOF software (v4.50, Leco Co., CA, USA), including baseline correction, smoothing, noise reduction, deconvolution, library searching, and peak area integration. Metabolites were identified by comparison of mass spectral similarity and Kovats retention index values against an in-house alkyl chloroformate derivative library, using a similarity score cutoff of > 70%.

Absolute metabolite concentrations (nmol/mL) were normalized to internal standards and fecal dry weight, and subsequently log2-transformed to improve normality. Metabolites detected in fewer than 50% of samples were excluded. Missing values, including values below the limit of detection, were imputed using half of the minimum observed value for each metabolite. No additional batch correction was applied, as QC samples demonstrated stable analytical performance throughout the acquisition sequence.

### Statistical analysis

Main statistical analyses were performed using R software. Continuous variables are presented as mean ± SEM. Paired comparisons were performed using paired Student’s t-tests for normally distributed variables and Wilcoxon signed-rank tests for non-normally distributed variables. Assumptions of multiple linear regression, including linearity, homoscedasticity, normality of residuals, independence, and absence of multicollinearity, were evaluated using residual plots and variance inflation factors. Linear regression analyses were conducted to determine the relationship between ΔVO₂peak and post-training VO₂peak and (i) baseline levels of proteins, fecal bacteria, and metabolites, and (ii) intervention-induced changes in proteins, fecal bacteria, metabolites, and (iii) VO₂peak, and baseline levels of proteins, fecal bacteria, and metabolites (*p* < 0.05). Covariates included in the regression models were age, baseline body weight, % fat mass, and baseline VO₂peak (for analyses of ΔVO₂peak). Multivariable linear regression models were specified a priori. Model 1 included age, lean mass and baseline VO₂peak; and Model 2 further included erythropoietin (EPO). EPO was selected a priori based on its established physiological relevance to oxygen transport and exercise adaptation. This sequential approach was used to evaluate the stability of estimates and potential confounding. Estimates are reported as coefficients with 95% CIs. Two-sided *P*<0.05 indicated statistical significance. To minimize model overfitting, the number of predictors included in each model was restricted considering the sample size and commonly recommended observations-to-predictor ratios. Multicollinearity was low for all predictors (all VIF < 1.2). Visual inspection of residual diagnostic plots demonstrated acceptable adherence to assumptions of linearity, homoscedasticity, normality of residuals, and absence of influential observations.

## Results

### Cohort characteristics

**Fig. 2:**
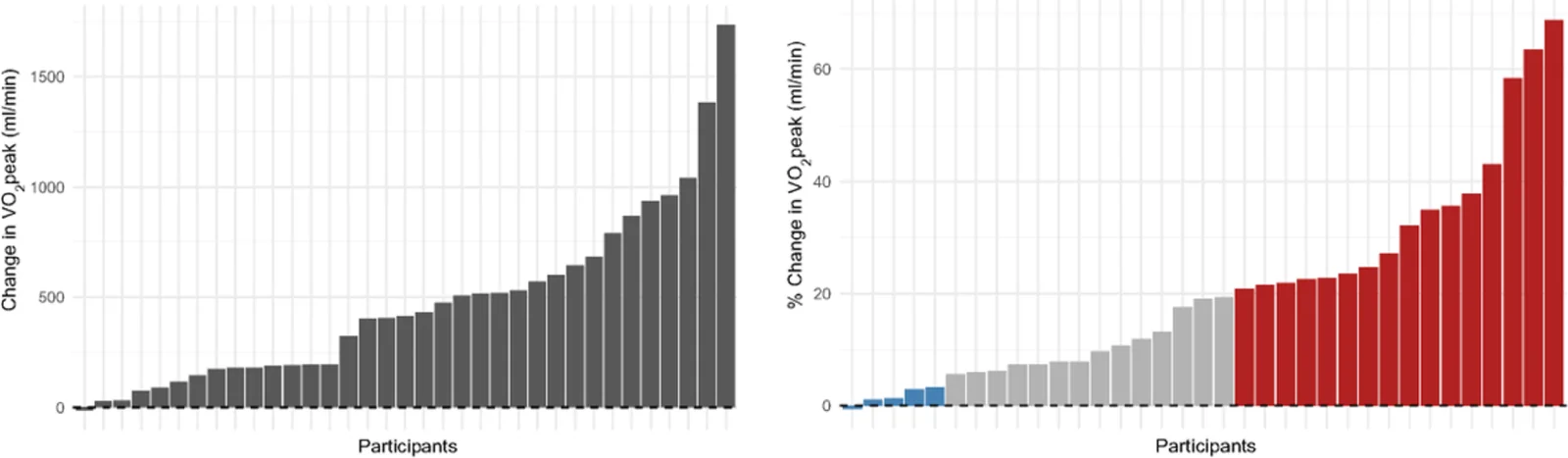
Individual responses to exercise training expressed as absolute and relative changes in VO₂peak. Bars represent individual participants ranked from the lowest to highest response. The left panel shows the absolute change in VO₂peak (ml/min), while the right panel shows the percentage change in VO₂peak following the intervention. In the right panel, blue bars indicate low responders (< 5% increase in VO₂peak), grey bars indicate moderate responders (5–20% increase in VO₂peak), and red bars indicate high responders (> 20% increase in VO₂peak) according to the predefined classification criteria

Clinical characteristics of thirty-five medication-naïve overweight and obese men with prediabetes enrolled in 12-week HIIT intervention are shown in Table 1. Notably, no significant differences were observed between the two cohorts included in this study (Supplementary Table 1). Following the intervention, mean VO₂peak increased by 0.47 L/min with individual responses ranging from 0 to 1.7 L/min (Fig. 2). This heterogeneity in VO₂peak response (∆VO₂peak) prompted us to explore the association between multi-omic changes and ∆VO₂peak.

**Table 1:** Clinical parameters in response to 12 weeks of high-intensity interval training (*N* = 35)

Variable	0-Week	12-Week	*p*-Value
Age (years)	40.66 ± 1.76	–	–
Weight (kg)	87.82 ± 2.10	85.95 ± 2.10	**< 0.001**
BMI (kg/m^2^)	30.14 ± 0.71	29.7 ± 0.72	**< 0.001**
Fat mass%	33.86 ± 0.79	31.98 ± 0.73	**< 0.001**
Lean Mass%	62.01 ± 0.64	65.28 ± 0.73	**< 0.001**
Fasting glucose (mM)	5.48 ± 0.08	4.94 ± 0.09	**< 0.001**
2 h OGTT glucose (mM)	8.21 ± 0.22	5.56 ± 0.31	**< 0.001**
Fasting insulin (µU/ml)	12.96 ± 1.55	9.11 ± 1.37	**< 0.001**
HOMA-IR	3.05 ± 0.36	1.93 ± 0.28	**< 0.001**
Triglycerides (mM)	2.32 ± 0.12	1.74 ± 0.13	**< 0.001**
Total cholesterol (mM)	5.35 ± 0.16	4.86 ± 0.14	**< 0.001**
HDL-c (mM)	1.17 ± 0.04	1.26 ± 0.04	**< 0.001**
LDL-c (mM)	3.59 ± 0.14	3.18 ± 0.11	**< 0.001**
Systolic blood pressure (mmHg)	132.35 ± 2.63	131.47 ± 2.52	0.686
Diastolic blood pressure (mmHg)	81.94 ± 1.56	80.32 ± 1.79	0.188
Resting heart rate (bpm)	76.03 ± 1.65	73.65 ± 1.57	0.058
VO_2_peak (ml/kg/min)	27.17 ± 0.64	33.33 ± 1.00	**< 0.001**
VO_2_peak (ml/min)	2380.78 ± 72.86	2852.33 ± 97.37	**< 0.001**

Data are presented as mean ± SEM. Abbreviations: BMI, body mass index; HDL-c, high-density lipoprotein cholesterol; HOMA, homeostatic model assessment for insulin resistance; LDL-c, low-density lipoprotein cholesterol; OGTT, oral glucose tolerance test; VO_2_peak, peak oxygen uptake, maximal oxygen consumption. Differences were assessed using a paired t-test or Wilcoxon signed-rank test, depending on data normality

### Baseline multi-omic signatures associated with VO₂peak trainability

**Fig. 3:**
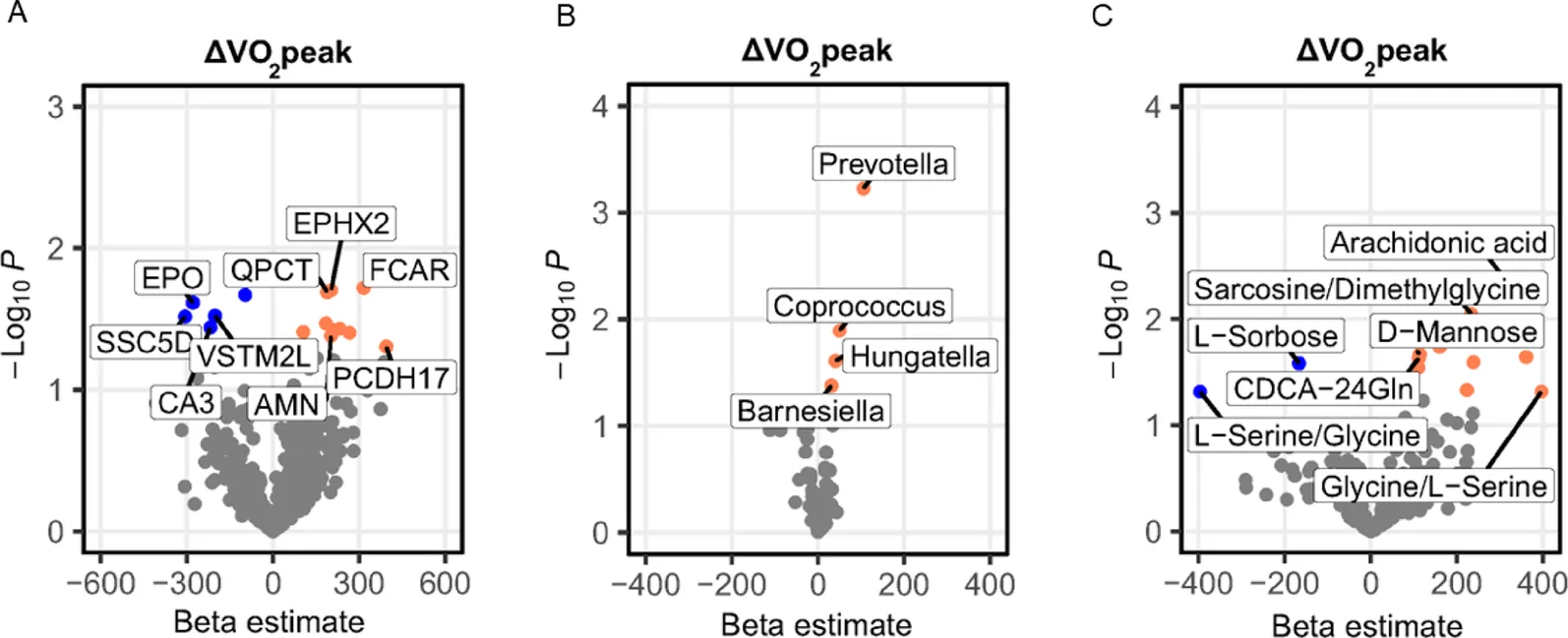
Baseline multi-omic signatures associated with changes in aerobic capacity (ΔVO₂peak). Baseline levels of proteins (**A**), fecal gut bacteria (**B**), and fecal metabolites (**C**) associated with changes in absolute VO₂peak (ΔVO₂peak) were identified using a linear regression model adjusted for age, weight, % fat, and baseline VO₂peak. Orange and blue dots indicate the direction of the association with metabolic outcomes, representing positive and negative associations, respectively, whereas grey dots indicate non-significant associations (*P* > 0.05)

We next examined the relationship between baseline multi-omic signatures and changes in absolute VO₂peak (ΔVO₂peak, ml/min) to identify potential factors influencing the physiological adaptations to HIIT. Regression analyses were adjusted for age, body weight, % body fat, and baseline VO₂peak. The final analysis included 688 proteins (352 from the cardiometabolic panel and 333 from the inflammatory panel and 3 included in both panels). We identified nine proteins positively associated with ΔVO₂peak, and five negatively associated (Fig. 3A). Among the positively associated proteins, soluble epoxide hydrolase (sEH)—encoded by the *EPHX2* gene—acts as a modulator of vascular tone by metabolizing epoxyeicosatrienoic acids, signaling molecules involved in vascular relaxation, into less active or inactive dihydroxy-eicosatrienoic acids. Among the negatively associated proteins, several were linked to erythropoiesis and inflammation. These include erythropoietin (EPO), the hormone regulating red blood cell production; scavenger receptor cysteine-rich family member with five domains (SSC5D), a member of the highly conserved scavenger receptor cysteine-rich superfamily of cell-surface and/or secreted proteins involved in immune signaling [19]; and V-set and transmembrane domain-containing 2-like (VSTM2L), a peripheral regulator of insulin action [20]. Regarding gut microbiota, baseline levels of bacterial genera associated with the production of short-chain fatty acids (SCFAs) were positively associated with ΔVO₂peak (Fig. 3B), including *Prevotella*, *Coprococcus* and *Hungatella*. Regarding fecal metabolomics, we identified, and fecal metabolomics identified ten metabolites positively associated with ΔVO₂max, and three negatively associated (Fig. 3C). Among the positively associated metabolites, arachidonic acid, an omega-6 polyunsaturated fatty acid, and a higher sarcosine-to-dimethylglycine ratio were linked to greater improvements in ΔVO₂peak. Conversely, lower baseline levels of valeric acid were associated with greater improvements in VO₂peak, suggesting a potential relationship between specific SCFA-related metabolic states and variability in adaptations to HIIT.

### Multi-omic changes associated with VO₂peak trainability

**Fig. 4:**
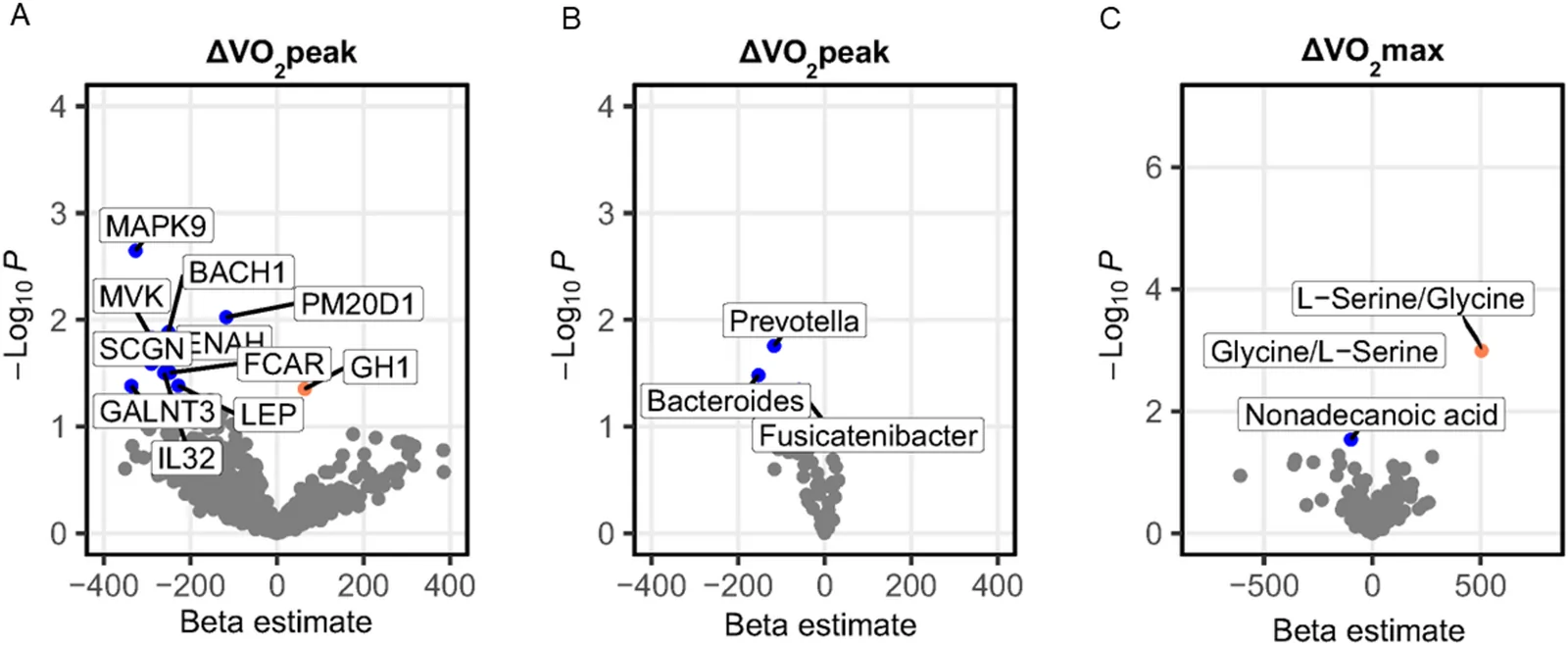
Multi-omic changes associated with changes in aerobic capacity (ΔVO₂peak). Changes in levels of proteins (**A**), fecal gut bacteria (**B**), and fecal metabolites (**C**) associated with changes in absolute VO₂peak (ΔVO₂peak) were identified using a linear regression model adjusted for age, weight, % fat, and baseline VO₂peak. Orange and blue dots indicate the direction of the association with metabolic outcomes, representing positive and negative associations, respectively, whereas grey dots indicate non-significant associations (*P* > 0.05)

We examined the relationship between multi-omic fold changes and ΔVO₂peak to identify potential mediators of VO₂peak adaptations to HIIT. We identified one exercise-induced protein that was positively associated with ΔVO₂peak and ten that were negatively associated (Fig. 4A). We observed that growth hormone (GH1) was positively associated with ΔVO₂peak. Among the negatively associated proteins, we identified mitogen-activated protein kinase 9 (MAPK9), a stress-activated kinase that regulates gene expression by modifying transcription factors [21], and BTB and CNC homology 1 transcription regulator (BACH1), a transcription factor that represses antioxidant genes [22].

Regarding gut microbiome, exercise-induced changes in three bacterial genera were negatively associated with ΔVO₂peak (Fig. 4B), including *Prevotella*,* Bacteroides and Fusicatenibacter*. In addition, fecal metabolomics revealed one three metabolites positively associated with ΔVO₂peak and two seven metabolites negatively associated (Fig. 4C). A greater increase in the L-serine/glycine ratio was linked to larger improvements in ∆VO₂peak, suggesting a potential shift with amino-acid-dependent energy pathways. In contrast, the odd-chain saturated fatty acid nonadecanoic acid (C19:0) was negatively associated with ΔVO₂peak, indicating possible lipidomic signatures reflecting less favorable adaptations to HIIT.

### Baseline multi-omic signatures associated with VO₂peak

**Fig. 5:**
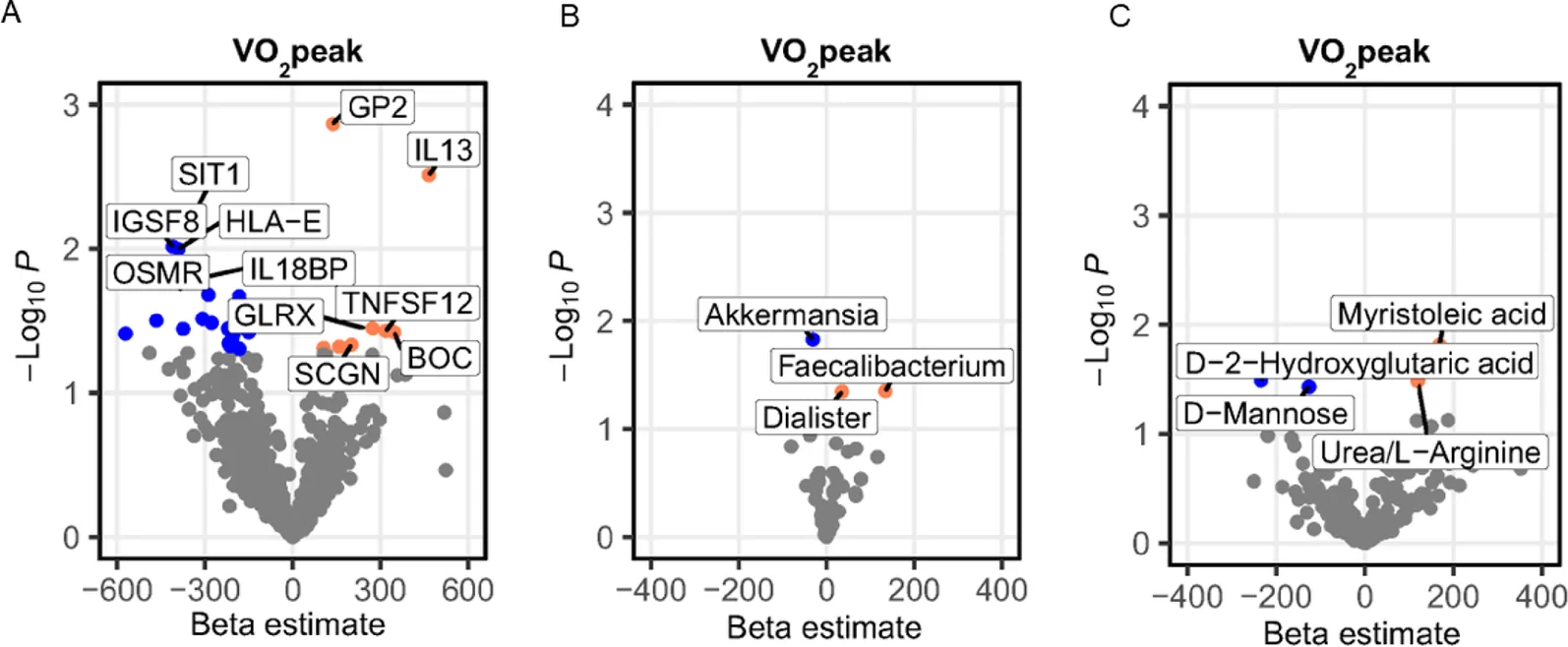
Baseline multi-omic signatures associated with aerobic capacity (VO₂peak). Baseline levels of proteins (**A**), fecal gut bacteria (**B**), and fecal metabolites (**C**) associated with absolute VO₂peak were identified using a linear regression model adjusted for age, weight, % fat. Orange and blue dots indicate the direction of the association with metabolic outcomes, representing positive and negative associations, respectively, whereas grey dots indicate non-significant associations (*P* > 0.05)

We next examined the relationship between baseline serum proteins and VO₂peak to explore factors associated with absolute VO₂peak using regression analyses adjusted for age, weight, % body fat. We identified eight proteins positively associated with VO₂peak, and twenty two five proteins showing a negative association (Fig. 5A). Notably, the protein with the strongest positive association (based on p value) with baseline VO₂peak was pancreatic secretory granule membrane major glycoprotein GP2 (GP2), a secreted protein expressed in zymogen granules of pancreatic acinar cells as well as in plasma membrane of intestinal M cells. Conversely, inflammatory markers, including Signaling Threshold-Regulating Transmembrane Adaptor 1 (SIT), Immunoglobulin Superfamily Member 8 (IGSF8) and Oncostatin M Receptor (OSMR) were negatively associated with VO₂peak. Regarding fecal bacteria and metabolites, two bacterial genera and three metabolite were positively associated with VO₂peak and one bacterial genera and two metabolites showed negative associations (Fig. 5B and C). Notably, *Faecalibacterium*, a bacteria genus associated with anti-inflammatory effects [23], showed the strongest positive correlations with VO₂peak, whereas D-2-Hydroxyglutaric Acid (D-2-HG), a metabolite associated with cancer, showed negative correlations with VO₂peak.

### Multiple linear regression model

In the clinical model (model 1), % lean mass emerged as the only baseline variable significantly associated with ∆VO₂peak together explaining 27% of the variance. We then assessed whether selected proteomic markers provided additional explanatory information beyond clinical variables. The inclusion of EPO (model 2) increased the adjusted R² from 0.27 to 0.39 (Table 2), which may indicate that the proteomic profile contributes to explaining interindividual differences in VO₂peak response.

**Table 2:** Multiple linear regression models predicting changes in absolute VO₂peak model fit statistics

Variable	Model 1	Model 2
Age	– 6.11 (95% CI – 18.33 to 6.11; *p* = 0.316)	– 5.54 (95% CI – 16.91 to 5.83; *p* = 0.328)
Lean mass	57.19 (95% CI 23.14 to 91.25; *p* = 0.002)	47.70 (95% CI 15.04 to 80.36; *p* = 0.006)
Baseline VO₂peak	– 0.12 (95% CI – 0.41 to 0.17; *p* = 0.401)	– 0.11 (95% CI – 0.39 to 0.16; *p* = 0.400)
EPO	−	– 299.58 (95% CI – 551.72 to – 47.44; *p* = 0.022)

Model Fit Statistics		
Statistic	Model 1	Model 2
R²	0.277	0.395
Adjusted R²	0.207	0.314
ΔR²	–	0.119
Residual SE	353.2	328.3
F-statistic	3.95	4.90
Model *p*-value	0.017	0.004

Abbreviations: CI, confidence interval; EPO, erythropoietin; Residual SE, residual standard error; VO₂peak, peak oxygen consumption

Values are presented as regression coefficients with 95% confidence intervals (CI) and corresponding p-values. Model 1 includes age, lean mass, and absolute VO₂peak. Model 2 additionally includes EPO. Statistically significant associations were considered at *p* < 0.05

## Discussion

This study examined how baseline multi-omic profiles and their changes relate to VO₂peak trainability, and how baseline multi-omic signatures are associated with VO₂peak. Twelve weeks of HIIT significantly increased mean VO₂peak in individuals with prediabetes, although the magnitude of improvement varied substantially across participants. Baseline levels of SCFA-producing genera, including *Prevotella. Coprococcus and Hungatella*, were positively associated with ΔVO₂peak. In contrast, baseline EPO levels were negatively associated with changes in VO_2_peak. In addition, a strong relation was observed between exercise-induced changes in GH1 and ΔVO₂peak, whereas exercise-induced changes in BTB and CNC Homology 1 showed a negative association with ΔVO₂peak. The clinical model explained 27% of the variance in ΔVO₂peak, which increased to 37% with inclusion of proteomic features such as EPO.

The response of the cardiovascular system to chronic exercise is regulated by the secretion of specific proteins known as “exerkines” [11]. It has been proposed that HIIT induces a complex organ crosstalk mediated by exercise-responsive factors secreted from skeletal muscle, adipose tissue, liver, gut, among other organs [24]. A well-documented exercise-responsive factor is EPO, the primary hormone regulating erythropoiesis. EPO increases during the first 1–2 weeks of exercise training in healthy individuals and then returns to baseline with continued training [25], whereas it may remain elevated after 12 weeks of HIIT in individuals with prediabetes [12]. Interestingly, participants in our cohort with elevated baseline EPO exhibited blunted VO₂peak responses, which may reflect a compensatory relationship with hemoglobin and hematocrit, as previously observed in individuals with hematological disorders [26] and in type 2 diabetes [27]. Furthermore, elevated basal EPO might also serve as an indicator of chronic systemic stress or inflammation-induced EPO resistance, which effectively constrains cardiovascular plasticity [28]. Furthermore, exercise-induced changes in BACH1, a repressor of heme oxygenase-1 (HO-1), were negatively correlated with ΔVO₂peak. HO-1 is a rate-limiting enzyme in heme catabolism that contributes to the regulation of erythropoiesis and red blood cell homeostasis [29, 30]. Central hemodynamic factors, including red blood cell mass and blood volume expansion, are fundamental adaptations to ET limiting changes in VO₂peak in healthy individuals [31]. Collectively, these findings raise the possibility that baseline hematological status, or exercise-induced changes in proteins regulating erythropoiesis, may constrain or facilitate improvements in aerobic capacity in individuals with prediabetes.

Recently, the gut microbiome has emerged as a potential modulator of exercise responses in individuals with prediabetes [16]. In addition, specific microbial genera have been associated with enhanced endurance performance and improved metabolism in elite athletes [32]. In mice, treatment with antibiotics for two weeks reduced endurance capacity compared to the control group [33]. Conversely, supplementation with *Bacteroides uniformis* increased endurance performance [34]. Our findings revealed that baseline microbiota may be associated with the changes in VO₂peak trainability. In this context, higher relative abundances of short-chain fatty acid (SCFA)-producing genera, including *Prevotella. Coprococcus and Hungatella*, were associated with greater ∆VO₂peak. Fecal SCFAs are increased in athletes compared to control subjects [35]. In mice, treatment with intracolonic infusion of SCFAs propionate improved running times compared to a control group, suggesting that SCFAs may increase aerobic performance [32]. Mechanistically, SCFAs may support energy production and enhance mitochondrial oxidative capacity, potentially influencing aerobic metabolism [36]. Alternatively, these microbially derived metabolites may act as distal exerkines that prime the cardiovascular system for HIIT-induced adaptations [11]. Altogether, these findings suggest a potential association between baseline microbiome composition and heterogeneity in VO₂peak trainability, particularly in metabolically compromised individuals, such as those with or at risk of developing T2DM.

Our study also highlights a strong relationship between exercise-induced changes in GH1 and ΔVO₂peak. GH1 is an exercise-responsive hormone released by the anterior pituitary gland in response to acute and chronic exercise [12, 37, 38]. Administration of GH1 reduced adipose tissue and increased lean body mass (LBM) in older individuals [39]. Notably, LBM and VO₂peak have been previously associated in men and women of Hans Chinese ethnicity [40] and women of Caucasian ethnicity [41], suggesting that GH1 may regulate ΔVO₂peak by affecting body composition in individuals with prediabetes. Consistent with previous reports [42], we found that participants with higher baseline LBM demonstrated greater improvements in VO₂peak following HIIT. While exploratory, these findings are consistent with the possibility that higher LBM may facilitate aerobic adaptations to training in individuals with prediabetes.

This study has several limitations. First, the relatively small sample size and restrictive inclusion criteria, including the inclusion of men only, limit the generalizability of the findings to women and other populations. In addition, the absence of a sedentary control group limits the ability to distinguish exercise-induced effects from time-dependent changes, biological day-to-day variability and technical error. Although participants were required to achieve a minimum attendance rate of 85% for study inclusion, comprehensive adherence data were not available for all individuals, limiting the evaluation of the potential influence of training compliance on the observed adaptations. Another limitation is the focus on obesity-associated insulin resistance, with limited consideration of other phenotypes, such as lean insulin-resistant individuals, who may exhibit stronger genetic contributions. The timing of post-intervention sampling may also represent a source of biological variability. Although a 48–72 h window following the final exercise session was selected to minimize acute exercise effects, it may still reflect differential recovery states across participants. Exercise-induced hematological adaptations may represent another source of biological variability, and the incorporation of direct cardiovascular measurements, including blood volume and cardiac parameters, may provide greater insight into the underlying molecular mechanisms. Finally, the use of nominal p-values without correction for multiple testing represents an important limitation, and the findings should therefore be interpreted as exploratory. Validation of the proposed model in an independent cohort is warranted. Future studies including larger sample sizes, multiple sampling time points, diverse ethnic groups, women, and sedentary control participants are needed to confirm these findings and further explore their potential clinical relevance.

In conclusion, the present study identified multi-omic signatures associated with VO₂peak and VO₂peak trainability in individuals with prediabetes. The identification of baseline proteomic and metagenomic signatures associated with VO₂peak trainability may facilitate the clinical implementation of personalized exercise interventions aimed at improving cardiorespiratory fitness in individuals with prediabetes.

## Supplementary Information

Below is the link to the electronic supplementary material.

Supplementary Material 1

Supplementary Material 2

## Data Availability

Olink raw proteomics data are provided in Table S2 from previous publication [9]. Any additional information required to reanalyze the data reported in this work paper is available from the lead contact upon request.
