# Supplementary material for: Multi-omics signatures of circulating factors associated with cardiorespiratory fitness adaptations in individuals with prediabetes
Source: Cardiovasc Diabetol. 2026 Jul 12;25:302. doi: 10.1186/s12933-026-03286-x (PMC13651112; doi:10.1186/s12933-026-03286-x)
Supplement: Supplementary file 1 [file 12933_2026_3286_MOESM1_ESM.docx]

**SUPPLEMENTARY MATERIALS**

Supplementary Figure S1. Flow diagram of the study...	2

Supplementary Figure S2. Baseline multi-omic signatures and exercise-induced multi-omic changes associated with post-training VO₂peak.	3

Supplementary Table 1. Clinical characteristics of participants in the discovery and validation cohorts	4

**Supplementary Figure S1. Flow diagram of the study.** In the discovery cohort, participants were randomized to either a sedentary group (n=19) or an exercise group (n=23). Following exclusion due to insufficient attendance (n=3), 20 participants were included for VO₂peak analysis and underwent proteomics, microbiome, and fecal metabolite analyses. In the validation cohort, participants were allocated to an exercise group (n=32). Following exclusion due to insufficient attendance (n=2), 15 participants were included for VO₂peak analysis and underwent proteomics analysis.

Abbreviations: OGTT, oral glucose tolerance test; VO₂peak, peak oxygen uptake.

A

B

C

D

E

F

**Supplementary Figure 2. Baseline multi-omic signatures and exercise-induced multi-omic changes associated with post-training VO₂peak.** Baseline levels of proteins (A), fecal gut bacteria (B), and fecal metabolites (C), as well as changes in proteins (D), fecal gut bacteria (E), and fecal metabolites (F), associated with post-training VO₂peak were identified using linear regression models adjusted for age, weight, body fat percentage, and baseline VO₂peak. Orange and blue dots indicate the direction of the association with post-training VO₂peak, representing positive and negative associations, respectively, whereas grey dots indicate non-significant associations (P > 0.05).

**Supplementary Table 1. Clinical characteristics of participants in the discovery and validation cohorts at baseline (0-week) and following the 12-week intervention.**

|  | **0-week** | | | **12-week** | | |
| --- | --- | --- | --- | --- | --- | --- |
|  | Discovery and validation Cohort (n=35) | Discovery Cohort (n=20) | p-Value | Discovery and validation (n=35) | Discovery Cohort (n=20) | p-Value |
| Age (years) | 40.66±1.76 | 40.84±2.85 | 0.956 |  | — | — |
| Weight (kg) | 87.82±2.10 | 83.13±2.29 | 0.138 | 85.95±2.10 | 81.61±2.25 | 0.166 |
| BMI (kg/m^2^) | 30.14±0.71 | 29.47±0.92 | 0.544 | 29.70±0.72 | 29.29±0.93 | 0.821 |
| Fat mass% | 33.86±0.79 | 36.28±0.99 | 0.064 | 31.98±0.73 | 33.94±0.92 | 0.102 |
| Lean Mass% | 62.01±0.64 | 60.88±0.91 | 0.316 | 65.28±0.73 | 63.25±0.85 | 0.075 |
| Fasting glucose (mM) | 5.48±0.08 | 5.61±0.08 | 0.273 | 4.94±0.09 | 4.96±0.09 | 0.703 |
| 2h OGTT glucose (mM) | 8.21±0.22 | 8.34±0.34 | 0.742 | 5.56±0.31 | 6.20±0.31 | 0.152 |
| Fasting insulin (µU/ml) | 12.96±1.55 | 12.33±2.28 | 0.644 | 9.11±1.37 | 9.30±2.30 | 0.683 |
| HOMA-IR | 3.05±0.36 | 2.88±0.51 | 0.657 | 1.93±0.28 | 1.91±0.46 | 0.683 |
| Resting heart rate (bpm) | 76.03±1.65 | 76.61±2.23 | 0.835 | 73.65±1.57 | 73.44±2.12 | 0.939 |
| VO_2_peak (ml/kg/min) | 27.17±0.64 | 26.29±0.75 | 0.375 | 33.33±1.00 | 31.42±1.11 | 0.209 |
| VO_2_peak(ml/min) | 2380.78±72.86 | 2181.63±79.00 | 0.070 | 2852.33±97.37 | 2567.36±120.45 | 0.073 |

Data are presented as mean ± SEM. P-values represent comparisons between the combined discovery and validation cohorts (n = 35) and the discovery cohort (n = 20) at each time point. BMI, body mass index; OGTT, oral glucose tolerance test; HOMA-IR, homeostatic model assessment of insulin resistance; VO₂peak, peak oxygen uptake.
